# The difference in hinge axis position determined by virtual versus conventional planning in orthognathic surgery

**DOI:** 10.1186/s12903-025-07222-5

**Published:** 2025-11-29

**Authors:** Jonas Rosenbusch, Moritz Kanemeier, Thomas Stamm, Claudius Middelberg, Jonas Q. Schmid

**Affiliations:** Department of Orthodontics, University of Münster, Albert-Schweitzer-Campus 1, Gebäude W 30, Münster, 48149 Germany

**Keywords:** Orthognathic surgery, Virtual surgical planning, Hinge axis, Mounting, Virtual articulator

## Abstract

**Background:**

The aim of the study was to compare the hinge axis position (Ax) determined by virtual (VSP) versus conventional orthognathic surgery planning (CSP).

**Methods:**

This retrospective cohort study included 320 (female/male = 204/116, median age 24.0 years) adult patients who received single- or two-jaw surgery originally planned using the CSP method and subsequently reevaluated using the VSP method. Three parameters ($\alpha$ = angle between the occlusal plane and reference plane, AxV = perpendicular distance between Ax and the occlusal plane, AxH = perpendicular distance between the upper incisor and AxV) for determining Ax were measured on cephalograms, both in VSP and CSP. Paired t-tests and Bland-Altman plots were used for statistical analysis.

**Results:**

Both methods differed significantly with a bias of -1.83$^\circ$ (95% CI -2.04 to -1.62) for $\alpha$, -0.24 mm (95% CI -0.41 to -0.08) for AxV and 0.46 mm (95% CI 0.27 to 0.65) for AxH, with negative values indicating an overestimation by the CSP method. All differences were within the range considered as clinically acceptable.

**Conclusions:**

There were statistically significant differences in all parameters; however, these differences were small and within the range generally considered to be clinically acceptable.

## Background

Determining the intercondylar hinge axis of the temporomandibular joint (TMJ) is an essential part of orthognathic surgery planning, whether it is virtual planning on the computer or conventional planning using an articulator. Determining the hinge axis requires that the mandible with its condyle is moved into a reproducible position within the fossa and thus into a reproducible position in relation to the skull. Years of research have shown that there is not just one ideal position of the condyles in the fossa, but that the reproducible position is the most relevant for therapy [1, 2]. Any deviation from this patient-specific position leads to disturbances in occlusion and errors in the planning of orthognathic surgery.

Posselt [3] has already shown that every patient has an initial rotational movement in the TMJ, a finding that has been confirmed several times by axiography in the 1990 s [4, 5]. The smaller this rotational movement, the more likely it is to be reproduced in the same position [5]. Articulators based on arbitrary hinge axis determination have become successful in oral rehabilitation [6, 7]. As an instrument for masticatory analysis and for the fabrication of fixed and removable dental prosthesis, the articulator was also predestined as a planning instrument for orthognathic surgery [8–10].

With the general availability of cone beam computed tomography (CBCT), virtual surgical planning (VSP) methods have emerged that no longer bear any resemblance to articulator planning. The anatomical visualisation and the possibility to perform virtual osteotomies appear to render the decades-old knowledge of plaster and impression materials, face bows, articulator developments, and 2D cephalometry obsolete. Existing VSP studies confirm a high degree of accuracy in the positioning of the jaw segments. The deviations published in the literature are below the size of 2 mm described by Proffit as clinically acceptable [11].

An exception to this, however, is the positioning of the condyle-ramus segment (CRS), which has a higher error rate in conventional planning [12], in VSP a with condylar positioning device [13] and also in VSP with mandibular patient-specific implants [14]. To date, the positioning accuracy of the CRS appears to be independent of the planning system utilized. Therefore, it is crucial to establish a clinical reference that ensures reproducible results both pre- and perioperatively. Based on clinical experience, we hypothesize that this reference is the initial open rotation (hinge axis) of the mandible induced by manual guidance.

The aim of the present study is, therefore, to compare the values of virtual arbitrary hinge axis determination to the conventional hinge axis positions in a large cohort of patients who underwent orthognathic surgery. The primary outcome measure is the difference between the virtual and conventional face bow based hinge axis determination. The secondary outcome is to generate skeletal type dependent normative values which could be incorporated into existing VSP protocols.

## Methods

The present investigation is a retrospective cohort study conducted at the Dental School of the University of Münster. Approval for the study was obtained from the Ethics Commission of the Medical Faculty (2021-120-f-S). All orthognathic patients included in the study were conventionally planned using the KD-MMS [15, 16] and subsequently operated on in the Department of Cranio-Maxillofacial Surgery.

Medical records of patients who received conventional surgical planning (CSP) have been screened. The inclusion criteria encompassed complete records including upper and lower mounted plaster casts for both the initial and the planned situation of patients who received LeFort I or bilateral sagittal split osteotomies (BSSO) as either single-jaw or two-jaw procedures. Any form of segmental osteotomies (e.g., two-piece maxilla, Delaire-Joos, Zisser), as well as distraction osteogenesis procedures and LeFort II or III surgeries were excluded. Due to the split-cast system of the KD-MMS all plaster casts could be re-mounted in the SAM 2P articulator (SAM Präzisionstechnik GmbH, Gauting, Germany) for measuring the hinge axis position.

### Virtual hinge axis determination

Before describing the conventional method, the virtual determination, which is described in a previous paper [17], should be briefly outlined as follows. In previous conventional plannings in our department, the position of the physical face bow was routinely marked on the patient’s facial skin with metal markers before a cephalogram was taken. A retrospective analysis of the cephalograms marked in this way revealed that four landmarks are sufficient to calculate the position of the axis-orbital plane (AOP), the location of the arbitrary hinge axis on this plane, and the position of the maxillary dentition relative to the AOP. The four key landmarks were:

- porion inferior,
- soft tissue nasion,
- upper incisor edge, and
- distobuccal cusp of the first upper molar. 

**Fig. 1:**
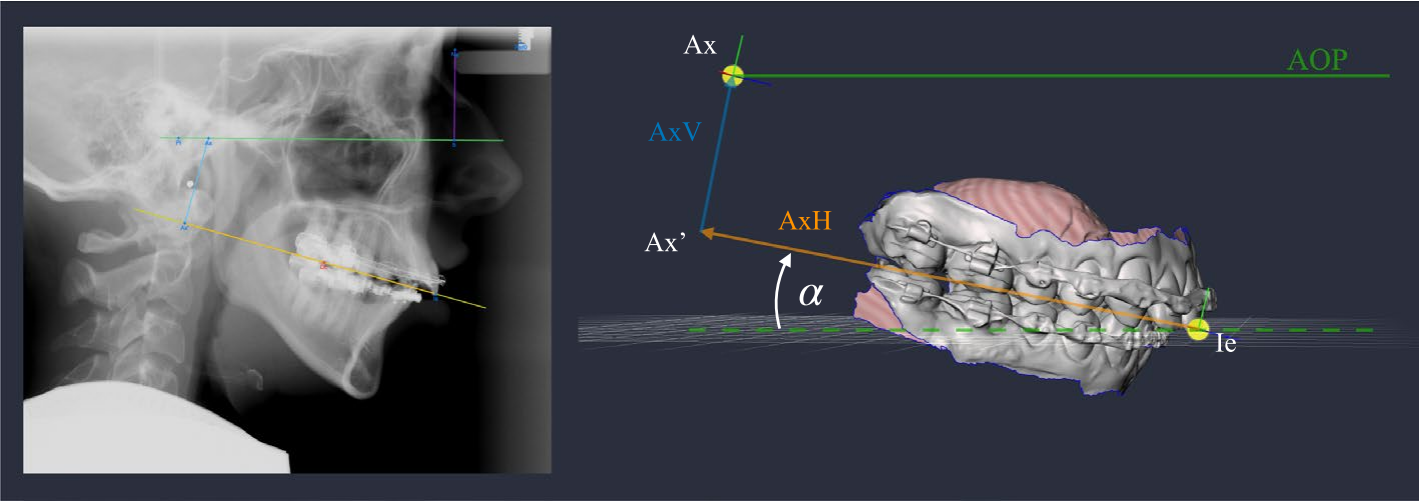
Projection of the virtual position of the facebow in the cephalogram using the landmarks porion inferior, soft tissue nasion, upper incisor edge, and distobuccal cusp of the first upper molar. This is used to calculate the AOP, the position of the hinge axis (Ax), the angle $\alpha$ between the occlusal plane and the AOP, the distance AxH as an extension of the occlusal plane distally, and AxV as the vertical distance from AxH to Ax

This allows constructing a virtual face bow, projecting it onto the cephalogram (Fig. 1), and provides three key values:

i) the angle $\alpha$ between the upper occlusal plane and the AOP,
ii) the vertical distance (AxV) between the virtual hinge axis (Ax) and its projection (Ax’) onto the occlusal plane, and
iii) the horizontal distance (AxH) between the upper incisor edge (Ie) and Ax’.

These values are sufficient to virtually replicate the position of the maxillary dentition mounted with a conventional articulator. Virtual orthognathic surgery planning using the Meshmixer software (Autodesk, Inc., San Rafael, USA) was performed with visible early contacts in the planned occlusion. For splint design, the mandible was rotated around the hinge axis in small increments (0.1$^\circ$) and Meshmixer’s clearance measurement function was applied to ensure that the splint achieved adequate thickness by verifying sufficient clearance between the dental arches.

### Conventional hinge axis determination

**Fig. 2:**
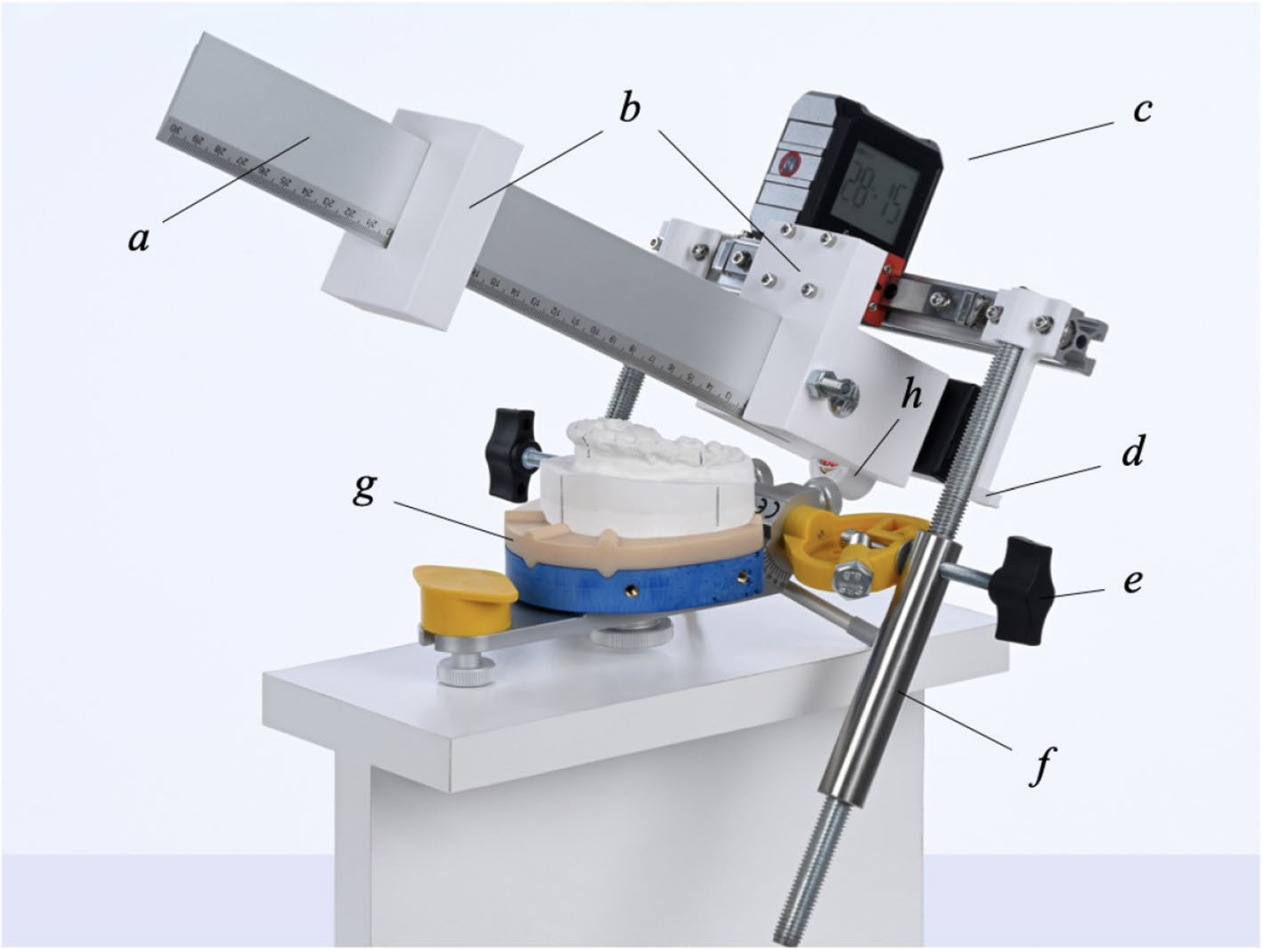
Customised measuring device attached to the condylar heads of the upper member of the SAM 2P articulator. (**a**) Aluminium ruler (elasto GmbH & Co. KG, Sulzbach-Rosenberg, Germany). (**b**) 3D-printed support for fixing the ruler, which allows displacement along the horizontal plane. (**c**) Digital spirit level (Shenzhen Dobiy Electronic Co., Ltd., Shenzhen, China) to measure the tilt ($\alpha$) of the occlusal plane. (**d**) 3D-printed calibrated support to indicate the plane of the laser (h). (**e**) Locking screw and lateral extension of the articulator’s axis. (**f**) Bilateral threaded rod for vertical adjustments. (**g**) Polymer split cast of the KD-MMS. (**h**) Cross-line laser (Picotronic GmbH, Koblenz, Germany) for visualization of the occlusal plane of the plaster cast dentition

The conventional measurement of the angle $\alpha$, the distances AxV, and AxH were performed on physically re-mounted plaster casts of surgical cases. The split cast plates of the KD-MMS, composed of industrially produced polymer, guarantee high dimensional stability over time. Therefore, an accurate reproduction of the initial planning situation with the original casts was possible in each selected case. To precisely determine the angle and distances on the articulator, a measuring device was fabricated that attaches to the articulator’s condylar heads (Fig. 2).

**Fig. 3:**
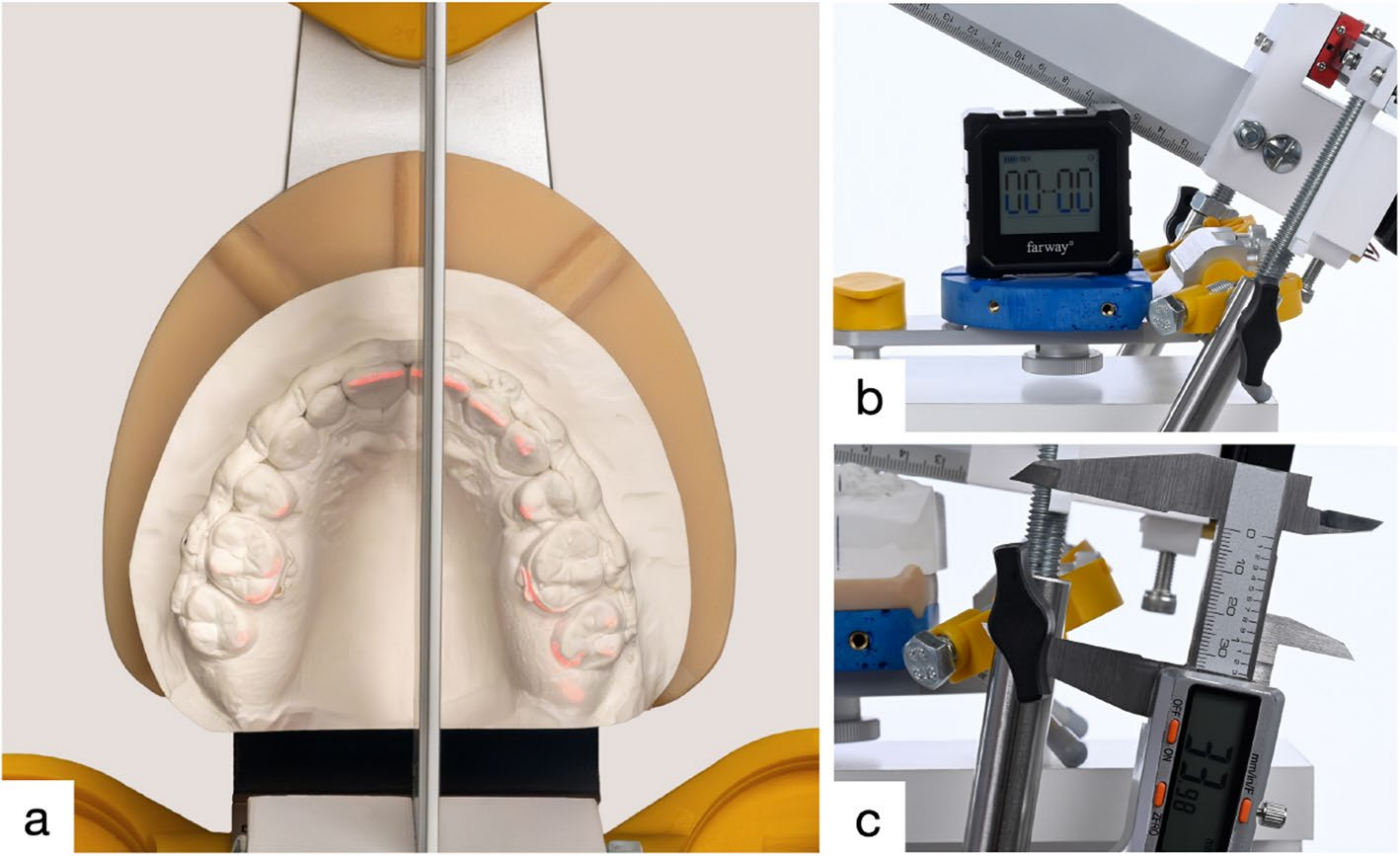
Details of the measurement process. **a** Scale positioned on the most proclined upper incisor edge and adjusted to the occlusal plane. **b** Digital spirit level set to zero on the upper split cast plate. **c** Measuring the perpendicular distance between the articulator’s condyle axis and the occlusal (laser) plane

Before placing the maxillary plaster cast, the digital spirit level was zeroed on the upper split cast plate and then attached to the ruler. This ensured that the ruler’s inclination was measured relative to the split cast plate (Fig. 3). After this the maxillary plaster cast was placed on the split cast, and the anterior part of the ruler was then fitted to the upper incisor edge. To consistently align with the most anterior upper incisor, the ruler can be moved along a linear guide in the horizontal plane.

Posteriorly, the ruler is adjusted to the level of the molars’ distobuccal cusps. This adjustment is achieved using a cross-line laser that projects a line into the dental arch at the level of the ruler. The final vertical adjustment is then made using the knurled screws. To fix this setting, the locking screws (e) are tightened. Subsequently, the following measurements can be carried out:

- Angle ($\alpha$): Measured by the digital spirit level placed on the newly positioned ruler.
- AxH: The origin of the ruler lies perpendicular to the hinge axis of the articulator (Ax). The distance between Ax and the upper incisor edge (Ie) can be easily read from the scale.
- AxV: The perpendicular distance from Ax to the occlusal laser plane, represented by a calibrated support along the threaded rod.

To calculate the measurement error, 30 randomly selected patients were used for repeated measurements. The virtual and conventional values were measured twice by one examiner (JR) at an interval of three months.

### Statistics

The statistical analysis of the data was carried out using R (version 4.3.2; [18]). An analysis of variance (ANOVA) was performed to assess group differences between the Angle Class and the vertical face type. To assess intrarater reliability for $\alpha$, AxV, and AxH in both conventional and virtual planning methods, 10% of the sample were randomly selected from the study cohort. These cases were each measured twice by the same examiner (JR) with a three-month interval between measurements. The intraclass correlation coefficient (ICC) for absolute agreement was calculated using a two-way mixed effects model as described by McGraw and Wong [19], implemented with the irr package [20] in R. Levels of reliability were interpreted according to the guidelines of Koo and Li [21]: poor reliability < 0.5, moderate reliability < 0.75, good reliability < 0.9, excellent reliability >0.9. A paired t-test was used to examine the analog and digital values for the presence of differences. Subsequently, potential differences were quantified using a Bland-Altman analysis [22], which was carried out using the blandr [23] package for R.

## Results

Virtual orthognathic surgery planning was introduced at the University of Münster in 2018. Therefore, cases from the years 2010-2018 were screened and 320 cases were included in this study. The intrarater reliability regarding the measurement of the mounting values ($\alpha$, AxV and AxH) was excellent for both planning methods (ICC >0.9).

### Baseline characteristics

Table 1 presents the baseline characteristics of the study cohort divided by gender, focusing on age and skeletal class. There was no significant difference in age between males and females (Mann-Whitney U test; p = 0.307). There was a statistically significant association between gender and the skeletal Angle class (${\chi }^2(2)~=~24.12$, $p~<~0.001$) and no association between gender and the vertical face type (${\chi }^2(2)~=~3.86$, $p~=~0.145$).

**Table 1:** Baseline characteristics of the study group divided by gender

		Age		Skeletal class				
	n	Median	IQR	I	II	III	High angle	Low angle
female	204	24	20.0–31.2	50	79	75	63	89
male	116	23	19.0–30.0	20	21	75	25	62
total	320	24	20.0–31.0	70	100	150	88	151

### Group differences

Group differences between the Angle Class and the vertical face type where evaluated with an ANOVA. There was no significant difference between the aforementioned groups. This means that neither skeletal Angle Class nor vertical face type had a significant influence on the measured variables. Therefore, the gender-specific distribution of the skeletal classes had no influence on the measured values and there was no need for a further group-specific analysis.

### Comparison of mounting values between methods

**Fig. 4:**
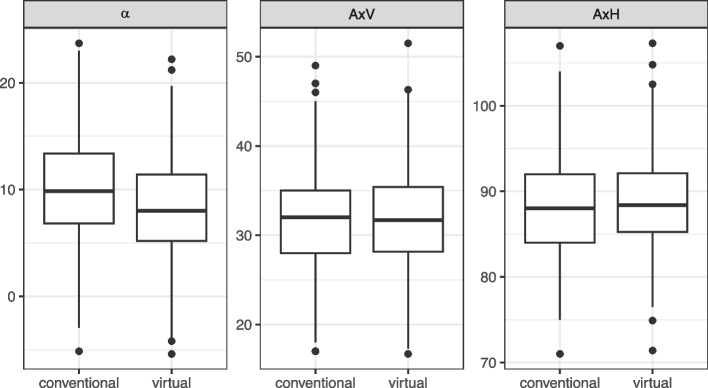
Boxplot of the mounting values separated by method (conventional and virtual surgical planning). The respective differences are statistically significant (paired t-test; $\alpha$ $p < 0.001$; AxV $p = 0.001$; AxH $p < 0.001$)

Table 2a and Fig. 4 present a comparative analysis between conventional (CSP) and virtual method (VSP) regarding hinge axis determination for the parameters $\alpha$, AxV, and AxH. For the angle $\alpha$, the VSP method showed a slightly lower mean value (8.4) compared to CSP (10.2), with an accompanying standard deviation that was nearly identical for both methods, which suggests a consistently lower $\alpha$ value with VSP, revealing a meaningful difference between the techniques (p < 0.001).

**Table 2a:** Mean (M), standard deviation (SD) and paired t-test statistics for the hinge axis in conventional (CSP) and virtual surgical planning (VSP)

	CSP			VSP			
	M$\varvec{\pm }$SD	Median	IQR	M$\varvec{\pm }$SD	Median	IQR	*p*-value
**Angle**$\varvec{\alpha }$	$10.2 \pm 4.9$	9.9	6.8 - 13.4	$8.4 \pm 4.8$	8.0	5.2 - 11.4	$< 0.001$
**AxV**	$32.1 \pm 5.9$	32.0	28.0 - 35.0	$31.8 \pm 5.7$	31.7	28.2 - 35.4	$< 0.05$
**AxH**	$88.3 \pm 5.8$	88.0	84.0 - 92.0	$88.7 \pm 5.6$	88.4	85.2 - 92.1	$< 0.001$

For the vertical distance AxV, the mean values for CSP and VSP were very close with similar standard deviations, indicating minimal variation between the two methods. However, the *p*-value was below 0.05, implying that even minor differences were statistically significant. In contrast, parameter AxH highlighted only marginal differences between the techniques, with almost identical mean values and very close standard deviations, showing a high level of similarity. The IQR for VSP are slightly narrower than those of CSP for all parameters, suggesting that VSP may offer more consistent outcomes. However, the difference appears to be clinically negligible.

**Fig. 5:**
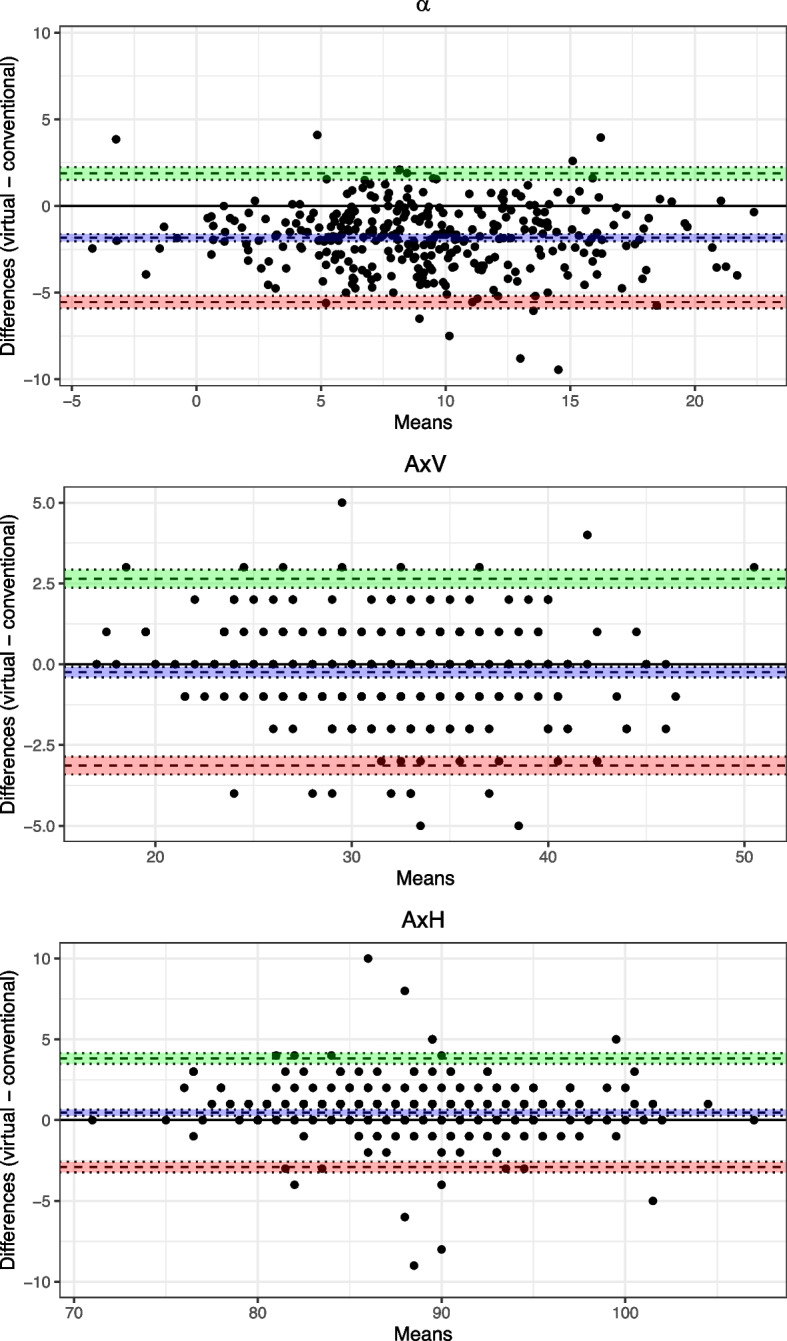
Bland-Altman plots for the mounting values ($\alpha$, AxV and AxH) determined by conventional and virtual surgical planning. Limits of agreement and bias are shown as dashed, black lines with 95% confidence intervals (green, red and blue areas)

The consistency between CSP and VSP measurements of the mounting values was evaluated using the Bland-Altman analysis. The Bland-Altman plots (Fig. 5) revealed no pattern or trends in the data. The values, indicated by $\alpha$, AxV, and AxH, reveal notable biases and limits of agreement (Table 3).

**Table 3:** Results of the Bland-Altman analysis of the mounting values ($\alpha$, AxV and AxH) measured by the conventional and virtual method (negative differences indicate an underestimation by the virtual method)

	Bias [95% CI]	ULoA [95% CI]	LLoA [95% CI]	*p*-value
$\alpha$	−1.83 [−2.04, −1.62]	1.88 [1.53, 2.24]	−5.54 [−5.90, −5.19]	$< 0.001$
AxV	−0.24 [−0.41, −0.08]	2.65 [2.37, 2.92]	−3.13 [−3.41, −2.86]	$= 0.003$
AxH	0.46 [0.27, 0.65]	3.82 [3.50, 4.14]	−2.90 [−3.22, −2.58]	$< 0.001$

*CI* Confidence interval, *ULoA* Upper limit of agreement, *LLoA* Lower limit of agreement

For $\alpha$, a bias of −1.83 degrees [95% CI: −2.04, −1.62] was observed, with upper and lower limits of agreement (ULoA and LLoA) at 1.88 [1.53, 2.24] and −5.54 [−5.90, −5.19], respectively ($p < 0.001$). This indicates a consistent underestimation by the digital method compared to the analog.

For AxV, the bias was −0.24 mm [95% CI: −0.41, −0.08], with ULoA at 2.65 [2.37, 2.92] and LLoA at −3.13 [−3.41, −2.86] ($p = 0.003$), suggesting a smaller systematic difference but relatively broad agreement limits.

Lastly, AxH showed a slight positive bias of 0.46 mm [95% CI: 0.27, 0.65], with ULoA and LLoA at 3.82 [3.50, 4.14] and −2.90 [−3.22, −2.58], respectively ($p < 0.001$), demonstrating a minor overestimation and widest agreement range among the three parameters.

In summary, there was a significant difference for all parameters, however the differences were small and within the range generally considered as clinically acceptable.

## Discussion

A recent meta-analysis has shown that the methodology for mandibular rotation used as a reference during VSP is still controversial [2]. In the VSP era rotating the lower jaw no longer means placing the condyles in the so called centric relation (CR). This position, which was considered therapeutically optimal, has historically moved from posterior to anterior, whereby the centric and posterior position is far more clearly supported by clinical studies and is therefore more evident than the anterior (cranio-ventral) position [24]. The CR as a theoretical, purely sagittal fixed position differs significantly from the lower jaw position set by the surgeon on the operating table. It can be assumed that manual manipulation of the mandible only produces an approximate rotation which underlines the difficulty or impossibility of assigning a geometric centre of rotation to the condyles. Instead of identifying one region for all patients, identifying the individual reproducible position seems to be clinically superior. This results in a patient-specific reference plane that can be defined by manual guidance and also has a high level of reproducibility with an error of 0.33 mm [25].

### Conventional versus virtual mounting

The Bland-Altman analysis provides essential insights into the agreement between the conventional and virtual measurement methods for the parameters angle $\alpha$, AxV, and AxH. The observed bias of −1.83 for angle $\alpha$ indicates that the virtual method tends to underestimate the angle measured compared to the conventional method. This is consistent with the findings of Quast et al. [26], and Zizelmann et al. [27] who reported an absolute difference between the occlusal plane and the used reference plane of $3.3 \pm 2.5$ degrees and $3.5 \pm 2.7$ degrees, respectively. The authors interpret the plaster cast mounting as too steep compared to values of a CBCT group. Compared to lateral cephalometric tracings even steeper values of $3.55 \pm 3.47$ degrees [28], $6.8 \pm 3.5$ degrees [29] and $7.8 \pm 4.2$ degrees [30] were published. With the limits of 1.88 and −5.54, our virtual method of occlusal plane angulation corresponds to the corrected values shown in the literature.

The bias of $-0.24 \, \text {mm}$ for the superior-inferior measurement AxV suggests a slight underestimation by the virtual method. The wider limits of agreement (from $-3.13 \, \text {mm}$ to $2.65 \, \text {mm}$) show that there is a noticeable variation among measurements, which is consistent with the findings of Kim et al. [31], who reported significantly lower values ($5.1 \, \text {mm} \pm 3.7 \, \text {mm}$) for the posterior height of the occlusal plane in VSP compared to CSP. The reason for the vertical differences may be linked to the clockwise over-rotated occlusal plane in CSP, as the centre of rotation lies within the plane, which affects both the anterior and posterior height. A further explanation may relate to the divergence of the estimated location of the meatus acusticus externus between CSP and VSP [31].

The anterior-posterior measurement AxH shows a slight positive bias of $0.46 \, \text {mm}$, indicating a minor overestimation by the VSP, with limits of agreement between $-2.90 \, \text {mm}$ and $3.82 \, \text {mm}$. Kim et al. [31] also found a more anterior position in VSP compared to CSP, although contradictory statements [32–34] are made in the literature. No values for the maxilla in the anterior-posterior direction can be found in the studies on clockwise rotation; however, Gateno et al. [30] simulated a conventionally planned maxillary advancement. Assuming a $10 \, \text {mm}$ advancement with a $12^\circ$ steeper inclination of the occlusal plane would result in a $1.5 \, \text {mm}$ smaller displacement. The larger value of AxH measured here, i.e. the distance between the hinge axis and the upper incisor edge, could, therefore, be an effect of the lower inclination of the occlusal plane in VSP.

Regarding the primary outcome, systematic errors in the physical facebow transfer and articulator mountings where found to cause deviations in the hinge axis determination between CSP and VSP. The direction and magnitude of these deviations are consistent with the error data presented in the literature and attest to the high accuracy of the virtual method employed [35].

Regarding the secondary outcome, the mounting values determined from CSP are not representative due to the inherent analogue errors confirmed here. The hypothesis that skeletal type-dependent normative values for arbitrary mountings can also be calculated from conventional planning must therefore be rejected. Presenting such values is therefore reserved for virtual methods [35].

### Strengths and Limitations

This study benefits from a large patient cohort and the direct comparison of conventional and virtual planning methods using standardized measurement protocols. However, its retrospective, single-center design may limit generalizability, and the study did not include long-term clinical follow-up. Future multicenter studies with prospective designs are warranted to confirm and extend these findings.

### Clinical implications

The presence of a patient-specific and constantly changing hinge axis during unguided opening movement, which complicates surgical planning, was recognised early on [36]. In contrast, guided movements exhibit a high degree of intra-individual reproducibility [25]. Despite this reproducibility, hand-guided mandibular rotations differ between awake, upright patients and anaesthetised, supine patients. In the former scenario, the neuromuscular component predominates, while in the latter, the ligament component is dominant. Findings from the literature suggest that this difference cannot be eliminated by any technology, whether virtual or conventional. Consequently, this discrepancy, which leads to inaccuracies in the operational realisation of planning, must be compensated for using alternative methods.

It has been observed that hand-guided neuromuscular opening rotation and ligamentous opening rotation are closely aligned, particularly if the surgeon also registers the mandibular position during clinical preparation for VSP. In this case, the CRS can adapt to positional errors postoperatively through physiological deformation. This physiological deformation must be facilitated by appropriate elastic plate systems [37]. As part of our protocol, the new position of the mandible should be stabilised for 4–6 weeks using a surgical splint and guiding elastics, allowing adequate time for physiological adjustment. In a previous study, it was demonstrated that this protocol, from planning to postoperative control after 6 weeks, achieved error sizes below the clinically accepted limit of two millimetres in translation and four degrees in rotation [38].

## Conclusions

The measured values between virtual and conventional determination of the hinge axis differ significantly. However, the differences were small and within the range generally considered as clinically acceptable. The differences correspond to the systematic errors shown in the literature for conventional articulator planning, both in size and direction. Therefore, the virtual method investigated in this study seems to be superior to conventional planning methods.

## Data Availability

The data presented in this study are available on reasonable request from the corresponding author.
